# The Importance of Fever as a Predictive Symptom for the Potency of Host's Monocytes to Release Pro- and Anti-Inflammatory Mediators

**DOI:** 10.1155/2008/450196

**Published:** 2008-03-12

**Authors:** Magdalini Kyriakopoulou, Anastasia Antonopoulou, Maria Raftogiannis, Fotini Baziaka, Thomas Tsaganos, Kyriaki Kanellakopoulou, Evangelos J. Giamarellos-Bourboulis

**Affiliations:** 4th Department of Internal Medicine, University of Athens Medical School, Attikon University Hospital, Athens 124 62, Greece

## Abstract

*Objective*. To clarify whether time lapsing from advent of fever as a first sign of sepsis may be indicative of the potency of monocytes for the release of pro- and anti-inflammatory mediators. *Methods*. Monocytes were isolated from blood of 51 septic patients and 9 healthy donors. Monocytes were incubated in the absence and presence of patients' serum and concentrations of tumour necrosis factor-alpha (TNF *α*), interleukin (IL)-6, IL-10, and malondialdehyde (MDA) were estimated in supernatants. Patients were divided into three groups: group A: *<*12 hours; group B: 12—24 hours, and group C: *>*24 hours between initiation of fever and blood sampling. *Results*. TNF *α* of supernatants of groups B and C was higher than controls, as also were IL-6 of A and C, IL-10 of A and B, and MDA of A. IL-6 of group A was increased after addition of patients serum. A negative correlation was found between time from initiation of symptoms and IL-6 of monocyte supernatants incubated in the presence of patients serum. Median IL-6 of survivors was higher than nonsurvivors. *Conclusion*. Monocytes are potent for the release of pro- and anti-inflammatory mediators within the first 24 hours upon advent of fever related to sepsis; serum stimulates further release of IL-6 within the first 12 hours.

## 1. INTRODUCTION

Despite the increase of knowledge on the mechanisms of pathogenesis of sepsis, its mortality remains high making sepsis the ninth cause of death in Northern Europe and in the United States [1]. The increased incidence of the septic syndrome has focused attention on various strategies for its management. Among these strategies, immunotherapies have been evolved. These comprised antibodies targeted against tumour necrosis factor-alpha (TNF*α*) and endotoxins, soluble receptors of TNF*α*, plasma hemodialysis, inhibitors of nitric oxide synthase, and activated protein C. Whereas most of them were successful when used in phase II clinical trials, they have either failed or proved problematic when applied in phase III clinical trials [2, 3]. Various hypotheses have been proposed to explain the failure of immunotherapies. The most important seems to be the lack of in-depth knowledge of the pathogenesis of sepsis [4]. Current theories of pathogenesis are based on the production of proinflammatory and anti-inflammatory cytokines after stimulation of blood monocytes by bacterial products and immunotherapies are often targeted against these mediators. However, in all clinical trials, therapies were administered upon fulfilment of certain criteria of enrolment without fully knowing the immunological status of the host on the time of start of any investigational product [4].

The importance of administration of immunotherapy on the time period when proinflammatory mediators reach their peak has been underscored by experimental studies [5]. To achieve that in the clinical field, it is mandatory to be able to correlate the presence of symptoms with the function of the immune system. Monocytes of septic patients are often deactivated, that is, they produce limited amounts of proinflammatory mediators when stimulated by bacterial products [6]. However, no data are available if monocytes of septic patients are capable to produce proinflammatory and anti-inflammatory mediators when transferred ex vivo. The present study attempted to provide such information and to correlate the ex vivo release of mediators from monocytes with the time lapsing from the initiation of fever as a first sign of sepsis.

## 2. PATIENTS AND METHODS

### 2.1. Characteristics of patients

Enrolment took place over the period January-September 2003 when the 4th Department of Internal Medicine was situated in “Sismanoglion” General Hospital of Athens. The protocol was approved by the Ethics Committee of the Sismanoglion General Hospital; written informed consent was taken from the patients or first-degree relatives. All consecutive admissions to the emergency department were eligible for the study.

Inclusion criteria were (a) the presence of fever defined as body temperature greater than 38°C being the first symptom of the underlying infection, (b) presence of one of the following underlying infections: lower respiratory tract infection, intrabdominal infection, or acute pyelonephritis, and (c) sepsis, severe sepsis, or septic shock. Exclusion criteria were: (a) HIV infection, (b) neutropenia defined as less 1000 neutrophils/ *μ*L, and (c) intake of corticosteroids at a dose greater than or equal to 1 mg/kg of equivalent prednisone for more than one month.

Lower respiratory tract infection was defined as the presence of all of the following [7]: (a) core temperature > 38°C, (b) new or increased cough, (c) new or increased purulent sputum production; and (d) new infiltrate on chest X-ray.

Intrabdominal infection was defined as the presence of all of the following [8]: (a) core temperature > 38°C, (b) pain on deep palpation, (c) radiological findings compatible with intra-abdominal infection, and (d) white blood cells > 12,000/ *μ*L or <4,000/ *μ*L or > 10% of band forms.

Acute pyelonephritis was defined as the presence of all of the following [9]: (a) core temperature > 38°C, (b) lumbar tenderness or radiological findings compatible with acute pyelonephritis, and (c) more than 10 white blood cells phf of centrifuged urine.

Sepsis was defined as the presence of an infection accompanied by at least two of the following [10]: (a) body temperature greater than 38°C, (b) respiratory rate higher than 20 breaths/min or *P*_CO__2_ < 32 mmH, (c) heart rate above 90 beats/min, and (d) white blood cells > 12,000 or <4,000/ *μ*L or more than 10% bands.

Severe sepsis was defined as sepsis complicated by the acute dysfunction of at least one organ due to an underlying infection, that is, the acute presentation of at least one of the following [10]:

- acute respiratory distress syndrome (ARDS): pO_2_/ FiO_2_ below 200 with diffuse bilateral shadows in lung X-ray;
- acute renal failure: urine production less than 0.5 mL/Kg body weight/h for at least two hours provided that the negative fluid balance of the patient was restored;
- metabolic acidosis: pH < 7.30 or any base deficit greater than 5 mEq/L and serum lactate at least more than 2x normal value;
- acute coagulopathy: platelet count < 100.000/ *μ*L or INR > 1.5.

Septic shock was defined as sepsis complicated with systolic blood pressure below 90 mmHg for more than one hour requiring the administration of vasopressors provided that the negative fluid balance of the patient is corrected [10].

Twenty five mL of whole blood were sampled from each patient after puncture of a peripheral vein under sterile conditions before initiation of any antimicrobial therapy; 20 mL were collected into a heparin-coated and sterile tube (Becton Dickinson, Coskeysville, Md, USA) and the remaining into a sterile tube. The latter was centrifuged and serum was kept at –70°C until assayed.

For each patient the following were recorded: age, sex, time interval between initiation of fever and blood sampling, as well as APACHE II score and outcome. The exact time of presentation of fever was provided by the patient’s history. Nonreliable patients were not considered eligible.

### 2.2. Laboratory techniques

For the isolation of peripheral blood mononuclear cells (PBMCs), heparinized venous blood was layered over Ficoll Hypaque (Biochrom, Berlin, Germany) and centrifuged. The separated mononuclear cells were washed three times with PBS (pH 7.2) and resuspended in RPMI 1640 supplemented with 2 mM of glutamine (Biochrom) in the presence of 100 U/mL of penicillin G and 0.1 mg/mL of streptomycin (Sigma-Aldrich, Miss, USA). After incubation for 1 hour at 37°C in 5% CO_2_, nonadherent cells were removed while adherent monocytes were washed three times with Hank's solution. Cells were then harvested by 0.25% trypsin/0.02% EDTA (Biochrom) and counted in a Neubauer plate after trypan blue exclusion. Their purity in monocytes was more than 95% as defined after incubation with the anti-CD14 mononuclear antibody at the fluorocolour FITC (Immunotech, Marseille, France) and analysis by the EPICS/XL flow cytometer (Beckman Coulter Inc., Miami, Fl, USA) using IgG-FITC-stained cells as negative controls.

Half of cells were treated with an ice-cold cell lysis buffer (50 mM HEPES, 0.1% CHAPS, 5 mM DTT, 0.1 mM EDTA, pH 7.4). After centrifugation for ten minutes at 10,000 g under 4°C, activity of caspase-3 was estimated in the cytosolic extract by an enzymatic chromogenic assay (BIOMOL Research Laboratories, Plymouth, Pa, USA). It was based on the rate of hydrolysis at 37°C of a substrate releasing p-nitroaniline overtime, as assessed by sequential photometry at 410 nm. The assay was also performed in the presence of a caspase-3 inhibitor. The activity of caspase-3 in cell extracts was expressed as pmol/min *⋅*10^4^ cells.

The remaining half of monocytes were distributed into two wells of a 12-well plate at a volume of 2.4 mL per well with RPMI 1640 supplemented with 2 mM of glutamine. They were incubated in the absence or presence of 100 *μ*L of the patient’ serum so that added serum represented 4.1% of the total well volume. After 24 hours of incubation at 37°C under 5% CO_2_, the plate was centrifuged and the supernatants were kept at –70°C until assayed. Monocytes isolated from nine healthy donors and incubated only in the absence of serum were applied as controls.

Concentrations of TNF*α*, interleukin (IL)-6, and IL-10 were estimated in serum and supernatants by an enzyme immunoabsorbent assay (Diaclone, Paris, France). The lower detection limits of the assay were 1.5 pg/mL for TNF*α*, 6.25 pg/mL for IL-6, and 12.5 pg/mL for IL-10. Their concentrations in monocyte supernatants were expressed as pg/10^4^ cells.

Lipid peroxidation was estimated in serum and supernatants by the concentration of MDA, as already described [11]. Briefly, a 0.1 mL aliquot of each sample was mixed to 0.9 mL of trichloroacetic acid 20% (Merck) and centrifuged at 12,000 g and 4°C for 10 minutes. The supernatant was removed and incubated with 2 mL of thiobarbituric acid 0.2% (Merck) for 60 minutes at 90°C. After centrifugation, a volume of 10 *μ*L of the supernatant was injected into a high-performance liquid chromatography system (HPLC, Agilent 1100 Series, Waldbronn, Germany) with the following characteristics of elution: Zorbax Eclipse XDB-C18 (4.6 × 150 mm, 5 *μ*m) column under 37°C; mobile phase consisting by a 50 mM K_3_PO_4_ (pH: 6.8) buffer and methanol 99% at a 60/40 ratio with a flow rate of 1 mL/min; fluorometric detection with signals of excitation at 515 nm and emission at 535 nm. The retention time of MDA was 3.5 minutes and it was estimated by a standard curve created with 1, 1, 3, 3-tetramethoxy-propane (Merck). All determinations were performed in duplicate. Concentrations were expressed as *μ*mol/mL of serum and *μ*mol/10^4^ cells of supernatant.

## 2.3. Statistical analysis

Results were expressed as median and 95% confidence intervals (CI) or as interquartile range (IQR). For the purposes of analysis, patients were divided into three groups according to the time interval between initiation of fever and blood sampling: group A: <12 hours, group B: 12 to 24 hours, and group C, >24 hours from start of fever. Comparisons between groups were done by Mann-Whitney U test with a Bonferroni correction; comparisons of yielded concentrations between absence and presence of serum by Wilcoxon’s test. Correlations between time interval from sampling and concentrations of cytokines were performed according to Spearman’s rank of order. Any value of *P* below .05 was considered as significant.

## 3. RESULTS

Over the period of enrolment, 54 patients were eligible. Fifty one were finally enrolled because only in them fever was the first symptom for underlying infection. Demographic characteristics of these patients are shown in Table 1. Sputum cultures failed to disclose any pathogen.

Thirteen patients belonged to group A, 16 patients to group B, and 22 patients to group C. Concentrations of TNF*α*, IL-6, IL-10, and MDA of serum in relation to that time interval are shown in Table 2. No differences were found between groups of time interval.

**Figure 1:**
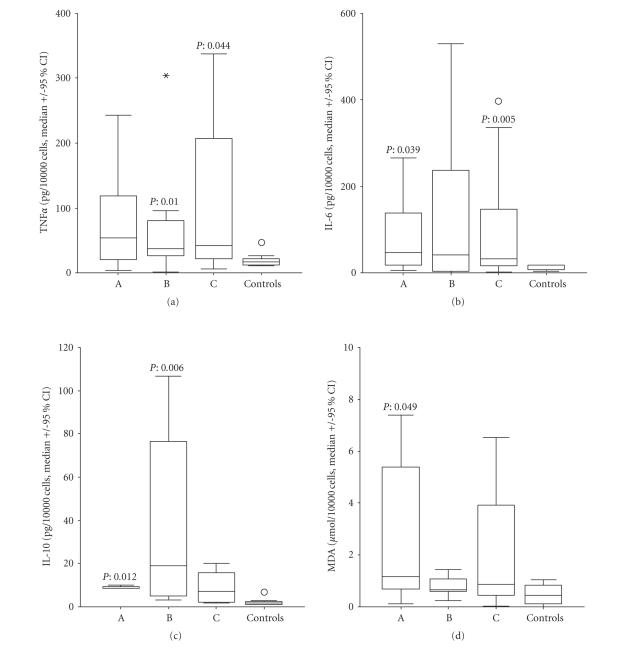
Ex vivo release of tumour necrosis factor-alpha (TNF*α*), IL-6, IL-10, and malondialdehyde (MDA) by monocytes of 51 patients with septic syndrome and nine healthy donors. Patients were divided into three groups depending on the time lapsing between blood sampling and advent of fever: group A: <12 hours group B: 12—24 hours, and group C: >24 hours. Asterisks denote outliers and circles denote extremes. *P* values refer to comparisons with controls.

Concentrations of TNF*α*, IL-6, IL-10, and MDA of monocyte supernatants without presence of patients’ serum in relation to the time interval of initiation of fever from sampling compared to controls are shown in Figure 1. TNF*α* of groups B and C was higher than controls, IL-6 of groups A and C higher than controls, IL-10 of groups A and B higher than controls, and MDA of group A higher than controls.

**Figure 2:**
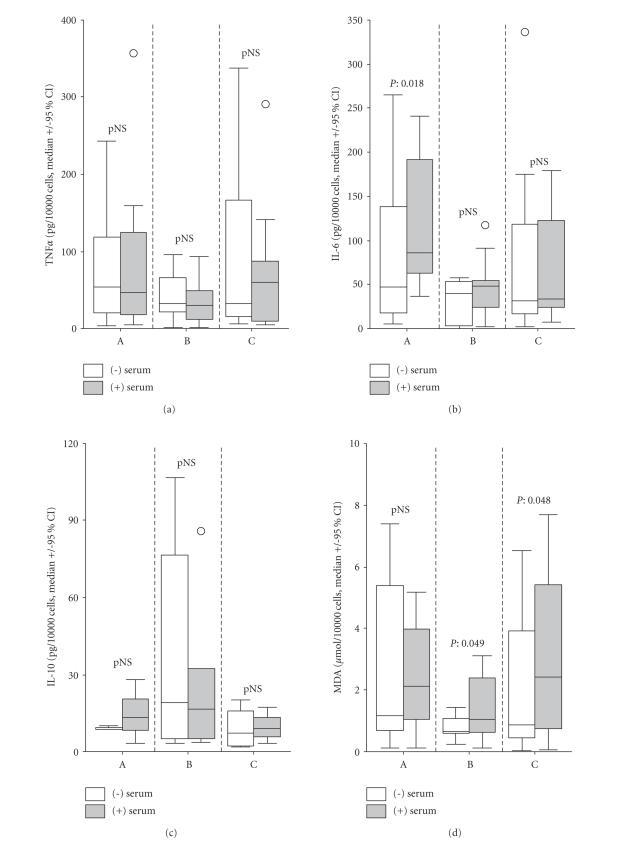
Effect of serum on ex vivo release of tumour necrosis factor-alpha (TNF*α*), IL-6, IL-10, and malondialdehyde (MDA) by monocytes of 51 patients with septic syndrome. Patients were divided into three groups depending of the time lapsing between blood sampling and advent of fever: group A: <12 hours, group B: 12—24 hours, and group C: >24 hours. Circles denote extremes.

The effect of serum on mediator release is shown in Figure 2. IL-6 of supernatants of monocytes isolated from patients of group A was increased after addition of patients’ serum (*P* = .018). That was the case for MDA of groups B (*P* = .049) and C (*P* = .048). IL-6 of supernatants of monocytes grown in the presence of patients’ serum was higher for group A compared to groups B (*P* = .037) and C (*P* = .048).

Median (IQR) activity of caspase-3 was 53.5 (295.5) pmol/min *⋅*10^4^ cells for group A, 200 (2020) pmol/min *⋅*10^4^ cells for group B, and 99.6 (1642) pmol/min *⋅*10^4^ cells for group C (pNS between groups of time).

**Figure 3:**
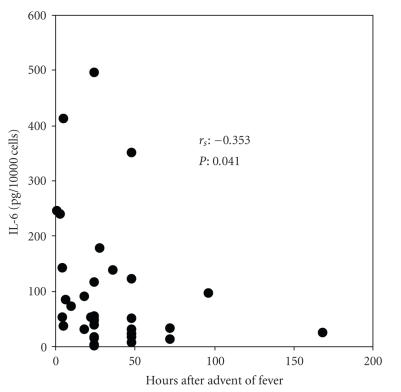
Correlation between time lapsing from advent of fever to blood sampling and concentrations of interleukin-6 (IL-6) released from monocytes of 51 septic patients incubated in the presence of patients’ serum.

A negative correlation was found between time from initiation of symptoms and IL-6 of monocyte supernatants incubated in the presence of patients’ serum ( *r*_s_: − 0.353, *P* = .041, Figure 3). No other significant correlation was found.

Median (IQR) IL-6 of supernatants of monocytes of survivors was 41.9 (149.2) pg/10^4^ cells compared to 1.9 (67.8) pg/10^4^ cells of nonsurvivors (*P* = .048). Respective values in the presence of serum were 51.6 (96.0) and 37.8 (30.0) pg/10^4^ (pNS). No differences were found in concentrations of TNF*α*, IL-10, and MDA of monocyte supernatants between survivors and nonsurvivors.

## 4. DISCUSSION

The immunological function of the septic patient admitted to the emergencies cannot yet be predicted with specificity despite the information provided by the existing serum markers. This is of prime importance since data derived from animal models underscore the need to know which immunological reaction takes place at which time so as to administer the appropriate type of immunotherapy [5, 12]. Although various studies exist providing in-depth knowledge about the sequence of events of the septic cascade in animals [4], there are various drawbacks for the human situation. The present study attempted to correlate the potency of monocytes of the septic host for the ex vivo release of pro- and anti-inflammatory mediators at various time intervals from the advent of fever in cases where fever was the initial symptom of the underlying septic process. To our knowledge, a similar attempt has never been described before.

Results revealed that monocytes of septic patients were most potent for the ex vivo release of TNF*α*, IL-6, IL-10, and MDA compared to healthy donors within the first 24 hours upon advent of fever (Figure 1). These findings signify that monocytes of septic hosts are still able to release a variety of mediators when put in an ex vivo milieu. Monocytes are probably primed in their septic environment for that production.

The pattern of release of mediators by monocytes is not ubiquitous since MDA is mainly secreted early, TNF*α* late, and IL-6 both early and late in relation to start of fever. Perhaps safe conclusions can be drawn only for IL-6 for two main reasons: (a) monocyte stimulation with patients’ serum yielded further release of IL-6 only for monocytes isolated within the first 12 hours from advent of fever (Figure 2), and (b) there is a negative correlation between time lapsing from start of fever and serum-stimulated release of IL-6 by monocytes (Figure 3).

What may be the underlying mechanism of induction of IL-6 by patients’ serum is not known and only hypothesis can be done. Serum cytokines do not seem responsible for that, since their levels do not differ between sera sampled at different time intervals from advent of fever (Table 2). Furthermore, caspase-3 activity of monocytes was similar in correlation to time lapsing from presence of fever, so it might not be hypothesized that endogenous inertia of monocytes was a culprit. Whatever might be the underlying mechanism, the clinical significance of that observation is that within the first 12 hours of fever, the monocytes of the septic host are embedded in an environment promoting the release of IL-6 (Figure 2). This environment seems to be a major determinant of the outcome of the septic patient. Despite the low number of deaths among the enrolled patients, ex vivo release of IL-6 was greater among survivors compared to nonsurvivors. That difference ceased to exist upon stimulation showing that the presence of serum elicited significant release of IL-6 from monocytes of nonsurvivors. This latter finding becomes of particular importance when considering data of animal studies. In experimental peritonitis in mice, prompt administration of imipenem could significantly decrease mortality with the sole exception of animals with very high serum concentrations of IL-6 [13].

The findings of the present study are in general agreement with those described in animal models of sepsis. Early sepsis in mice is accompanied by high serum concentrations of both pro-inflammatory and anti-inflammatory cytokines as opposed to low serum concentrations found in late sepsis [14]. On the same context, ex vivo release of TNF*α*, IL-6, and IL-10 by monocytes of the septic host was greater within the first 24 hours upon advent of fever.

In conclusion, our data revealed that fever might constitute a symptom predictive of the immune function of the septic host. Monocytes are potent for the release of pro- and anti-inflammatory mediators within the first 24 hours upon advent of fever; serum of patients stimulates further release of IL-6 within the first 12 hours. These findings are in general agreement with both experimental and clinical results that, as antibiotics, should be administered as early as possible in the septic host [15, 16], the same may be necessary for immunomodulatory treatment.

## Figures and Tables

**Table 1:** Demographic characteristics of 51 patients with septic syndrome enrolled in the study.

	Sepsis	Severe sepsis	Septic shock
Number	38	10	3
Male/Female	15/23	3/7	0/3
Age (mean ± SD)	59.8±24.8	73.6±12.4	78.0±7.0
APACHE II score (mean ± SD)	4.7±4.6	12.9±6.3	17.8±7.5
White blood cells(/*μ*l, mean ± SD)	12800.9±5366.5	16367.0±4734.3	13856.7±2511.7

Underlying infection [no. (%)]			
Lower respiratory tract infection	8 (21.1)	4 (40.0)	0
Intrabdominal	12 (31.6)	2 (20.0)	2 (66.7)
Acute pyelonephritis	18 (47.4)	4 (40.0)	1 (33.3)

Bacteremia [no. (% all enrolled patients)]			
*Providencia stuartii*	—-	2 (20.0)	—-
*Escherichia coli*	4 (10.5)	—-	—-

Positive urine cultures (>10^5^ cfu/ml) [no. (%)]			
*Escherichia coli*	16 (42.1)	3 (30.0)	1 (33.3)
*Pseudomonas aeruginosa*	2 (5.3)	1 (10.0)	—-
*Providencia stuartii*	—-	1 (10.0)	—-

Administered antimicrobials [no. (%)]			
2nd generation cephalosporin	11 (28.9)	1 (10.0)	—-
2nd generation cephalosporin + metronidazole	10 (10.5)	2 (20.0)	—-
Ceftriaxone + macrolide	8 (21.1)	4 (40.0)	—-
Piperacillin/tazobactam + vancomycin	0	3 (30.0)	3 (100)
Amplicilln/sulbactam	9 (23.7)	—-	—-

Death (%)	2 (5.3)	1 (10.0)	0 (0)

**Table 2:** Serum concentrations of tumour necrosis factor-alpha (TNF*α*), IL-6, IL-10, and malondialdehyde (MDA) of 51 septic patients in relation to the time of blood sampling from advent of fever.

	Group A (*n* = 13) <12 hours	Group B (*n* = 16) 12—24 hours	Group C (*n* = 22) >24 hours
		Median (IQR)	
TNF*α* (pg/mL)	9.88 (9.40)	7.23 (7.54)	7.64 (4.11)
IL-6 (pg/mL)	94.3 (225.1)	65.1 (179.2)	55.4 (120.5)
IL-10 (pg/mL)	<12.5	<12.5	<12.5
MDA ( *μ*mol/mL)	0.80 (11.30)	2.87 (9.40)	4.75 (10.05)
